# Insights into the regulation of mRNA translation by scaffolding proteins

**DOI:** 10.1042/BST20241021

**Published:** 2024-12-06

**Authors:** Madeleine R. Smith, Guilherme Costa

**Affiliations:** Wellcome-Wolfson Institute for Experimental Medicine, Queen's University, Belfast BT9 7BL, U.K.

**Keywords:** cellular localization, gene expression and regulation, molecular scaffolds, RNA localization, translation

## Abstract

Regionalisation of molecular mechanisms allows cells to fine-tune their responses to dynamic environments. In this context, scaffolds are well-known mediators of localised protein activity. These phenomenal proteins act as docking sites where pathway components are brought together to ensure efficient and reliable flow of information within the cell. Although scaffolds are mostly understood as hubs for signalling communication, some have also been studied as regulators of mRNA translation. Here, we provide a brief overview of the work unravelling how scaffolding proteins facilitate the cross-talk between the two processes. Firstly, we examine the activity of AKAP1 and AKAP12, two signalling proteins that not only have the capacity to anchor mRNAs to membranes but can also regulate protein synthesis. Next, we review the studies that uncovered how the ribosome-associated protein RACK1 orchestrates translation initiation. We also discuss the evidence pointing to the scaffolds Ezrin and LASP1 as regulators of early translation stages. In the end, we conclude with some open questions and propose future directions that will bring new insights into the regulation of mRNA translation by scaffolding proteins.

## Introduction

The intracellular space is a highly complex environment where synchronised and simultaneous molecular processes determine cell function and behaviour. To ensure efficiency and fidelity of these mechanisms in such crowded settings, scaffolding proteins target molecular assemblies to subcellular compartments. This way, localised interactions between components are facilitated in a spatiotemporal manner. Not surprisingly, scaffolds can play essential roles in development and their disruption may underlie pathological conditions, from ischaemic stroke [1] and cancer [2], to Alzheimer's disease [3]. To this date, more than 300 proteins have been classified as scaffolds and they are mostly, but not only, understood as platforms in which signalling takes place. In this context, they may even form signalosomes, large complexes with the potential to bring together multiple proteins in signalling islands for pathway cross-talk and local signal amplification [4]. Other biological roles have been associated to scaffolding proteins beyond signalling. This class of proteins has also been shown to play roles such as cytoskeletal remodelling, regulation of endocytosis and importantly, mRNA translation. Despite the modest number of examples demonstrating the latter, strong evidence supports the concept that protein production outputs can be controlled by scaffolding protein activity. Given their ability to compartmentalise components of translational complexes, scaffolds can regulate mRNA translation in a regionalised manner and thus, contribute towards spatial control of gene expression. This phenomenon, which entails subcellular localisation of mRNAs and translational regulators, underpins fundamental biological processes and has been documented in many cell types and organisms [5]. For example, while mRNA localisation to the leading edge of endothelial cells is believed to contribute towards endothelial cell morphology during angiogenesis [6], protein synthesis at the outer mitochondrial membrane has critical metabolic implications [7]. Much like the consensual understanding that scaffolds enhance mechanistic efficiency, compartmentalised translation supports protein targeting and function. From this perspective, subcellular mRNA translation is believed to increase the local concentration of newly synthesised proteins or even to facilitate the assembly of subunits encoded by co-localised transcripts [8]. Thus, scaffold-mediated regulation of translation and spatial control of gene expression are inherently intertwined concepts. Due to the extraordinary versatility of scaffolding proteins, scaffold-dependent mRNA translation is not independent from other mechanisms converging on these anchoring platforms. In fact, extremely intricate and interdependent processes may hinder the dissection of unique pathways controlling translation.

In this mini-review, we cover findings that have proposed how scaffolding proteins co-ordinate protein production and their potential implications in spatial biology. We discuss proteins that were firstly implicated in processes other than translation but are now understood to regulate it via the binding to mRNA or through the interaction with other proteins. Lastly, we present gaps in knowledge and hypotheses related to these scaffolds, as well as some open questions in the field.

## Scaffold-mediated control of protein synthesis

### Through mRNA binding and localisation

The binding of scaffolding proteins to mRNA templates, and the consequent influence on translation outputs, is an expanding concept that is well exemplified by the activity of A-kinase anchoring proteins (AKAPs). These proteins compose a large family of scaffolds that control a multitude of biological processes through the anchoring of many signalling-related enzymes to specific subcellular compartments, enhancing the efficiency of localised signal transduction [9]. As the family name suggests, AKAPs are most studied for their function tethering protein kinase A (PKA) to microdomains [10]. PKA is a holoenzyme composed of catalytic and regulatory subunits that dissociate in the presence of cyclic adenosine monophosphate (cAMP) to elicit the phosphorylation of target substrates. All AKAPs interact with PKA through their conserved 5-turn amphipathic helix, a 14–18 amino acid structure that docks the regulatory subunits of PKA [11,12]. Some AKAPs can also act as RNA binding proteins (RBPs), a feature with the potential to render these scaffolds as direct translational regulators of their target transcripts. AKAP1 and AKAP12 are two members known to display such functional properties, although to different extents.

**Figure 1.**
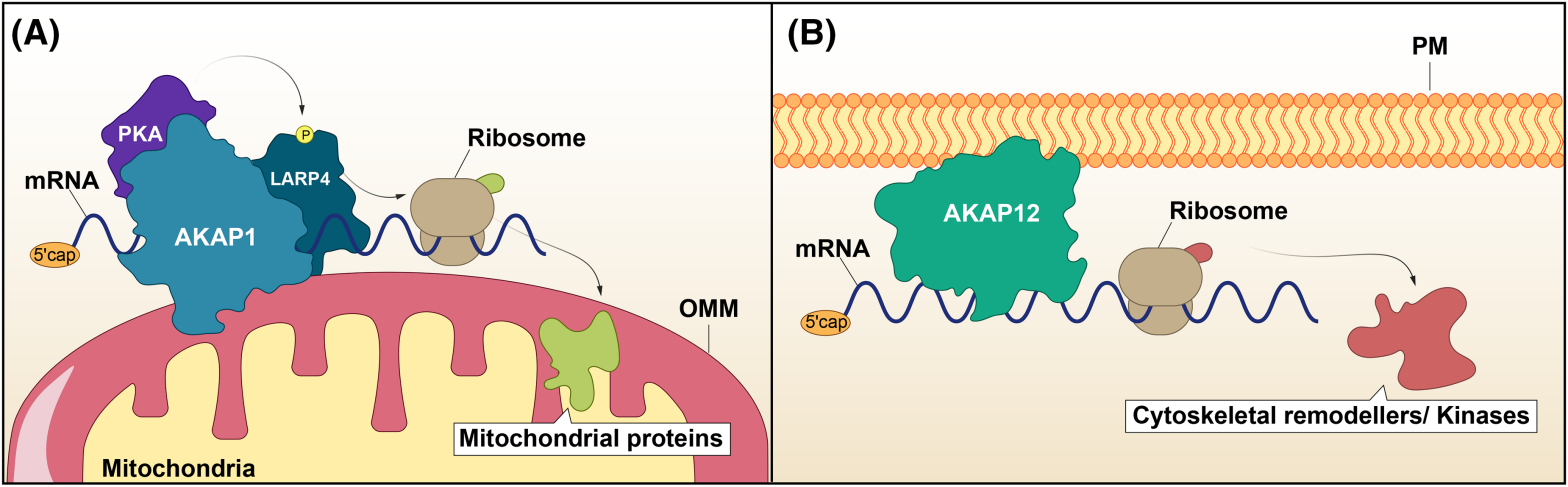
AKAP-dependent translation. (**A**) AKAP1 docks mRNAs, LARP4 and PKA to the outer mitochondrial membrane (OMM) where proteins necessary for mitochondria function are translated. (**B**) AKAP12 anchors mRNAs to the plasma membrane (PM) and is necessary for efficient production of proteins with cytoskeletal remodelling and kinase activity. P, phosphorylation.

AKAP1, originally named S-AKAP84, was firstly identified in association with mitochondria of germ cells [13]. Follow up studies described AKAP1 as a protein containing a K-homology (KH) RNA binding domain in its C-terminus, and later shown to harbour N-terminal targeting domains responsible for its localisation to the outer mitochondrial membrane or the endoplasmic reticulum [14–16]. Notably, kinases and phosphatases alter the phosphorylation of the KH domain and consequently, determine AKAP1-RNA interactions. Loss of Serine phosphorylation within a PKA phosphorylation consensus site found in the KH domain reduces its RNA-binding affinity but enhances the interaction with the phosphatase PP1 [17,18]. This behaviour suggested a ‘switching’ model in which AKAP1 could alternate between mutually exclusive PP1- and RNA-bound states [18]. Altogether, these observations have laid the foundations for following studies demonstrating AKAP1-controlled mRNA translation as a mechanism underpinning aspects of mitochondria biology and metabolism [19]. AKAP1 interacts with and targets La Ribonucleoprotein 4 (LARP4) to the mitochondria (Figure 1A) [20], an RBP with several roles in RNA biology including stimulation of translation and highly expressed in metabolic tissues [21]. This interaction could indicate the establishment of a local regulatory site for protein synthesis, an idea further supported by recent profiling studies demonstrating that one quarter of all LARP4-bound mRNA species encode mitochondria-associated proteins [22]. Similar mechanisms were described in *Drosophila* embryos and thus, AKAP1 roles in mRNA anchoring and translation of its targets are believed to be highly conserved [23]. Interestingly, LARP4 is a substrate for PKA in an AKAP1-dependent manner [20], implying that compartmentalised signalling events may impact protein synthesis at the mitochondria surface. In adipocytes, AKAP1 binds the 3′ untranslated region (UTR) of the mRNA encoding the hormone-regulated lipoprotein lipase (LPL) and supresses its translation. This inhibitory effect on *LPL* mRNA can be relieved by elevating cAMP levels with exposure to epinephrine, a major determinant of lipid biology [24,25]. The regulation of Steroidogenic Acute Regulatory Protein (STAR) production is another example illustrating the remarkable ability of AKAP1 to link hormone signalling with protein synthesis. In this case, AKAP1 co-targets *STAR* transcripts and PKA to the mitochondria of Leydig and adrenocortical carcinoma cells. Once at its destination, local translation of *STAR* mRNA is promoted by cAMP signalling downstream of the steroid intermediate 22R-hydroxycholesterol [26,27]. The plethora of metabolic-related targets was expanded by Gabrovsek et al. [20] who used bacterial-purified versions of AKAP1 as baits to pull-down mRNAs from Leydig cells extracts. In this study, AKAP1 preferentially bound transcripts encoding metabolic proteins, including mitochondria-associated enzymes belonging to the tricarboxylic cycle and the electron transport chain. Remarkably, depletion of AKAP1 resulted in the dysfunction of these two coupled processes and in the concomitant reduction of newly translated proteins associated with mitochondria [20]. Such dramatic effects in the cell's energy powerhouse are likely to underlie the pathological phenotypes described in studies involving varied AKAP1 loss-of function models [28]. The dissection of mechanisms implicating AKAP1 in the modulation of energy production consolidated the relevance of this scaffolding protein in mitochondria biology. However, exactly how AKAP1-dependent processes are shaped by cAMP second messenger activity, or any other signalling pathways is not exactly clear.

The combined development of biochemical technologies and omics tools is reshaping protein classification and helps expanding the knowledge on the phenomenal potential of scaffolding proteins. This is the case for global profiling studies of RNA-bound proteomes that discovered the unappreciated capacity of vast numbers of proteins to interact with RNAs, many of which had been traditionally studied as proteins with scaffolding properties [29–31]. In an effort to identify novel RBPs present in plasma membrane-rich protrusions formed at the leading edge of motile endothelial cells, we recently reported the ability of AKAP12 to bind mRNAs [32]. AKAP12, also known as Gravin, was firstly isolated from patients with the autoimmune disorder myasthenia gravis [33]. Subsequently, AKAP12 has been described as a highly disordered scaffold that anchors multiple signalling proteins to the cell periphery, many of which are critical re-modellers of Actin structures and ultimately control cellular processes such as migration, cytoskeleton reorganisation and adhesion [34,35]. As well as binding to PKA through its dedicated domain, AKAP12 interacts with a plethora of signalling partners, such as protein kinase C (PKC), the β2-adrenergic receptor, phosphodiesterase-4, calmodulin and the non-receptor tyrosine kinase Src. We refer readers to reviews detailing the physiological and pathological implications of such interactions [34,36]. An N-terminal myristoylation site, polybasic domains and a calmodulin binding domain have all been implicated in targeting AKAP12 to the plasma membrane, whereas a filamentous (F)-actin binding domain anchors the protein to the cytoskeleton [37–39]. Despite the lack of classical RNA binding motifs, we demonstrated that AKAP12 preferentially binds mRNAs encoding regulators of F-actin and proteins with kinase activity, some with Actin remodelling roles as well (Figure 1B). In the absence of AKAP12, one of the mRNA targets was less abundant in the plasma membrane and showed reduced association with polysomes [32]. Considering our findings and prior work by Benz et al. [40] implying binding of ribosomal proteins and many RBPs to AKAP12, we proposed a novel function for this scaffold in posttranscriptional control of gene expression [32]. The pivotal role of AKAP12 in the establishment of a signalosome at the cell periphery is thus likely to go beyond the simple anchoring of signalling proteins. A much more complex scenario is emerging, in which the localised regulation of protein synthesis could influence local remodelling of the membrane-associated Actin cytoskeleton. Of note, many translational proteins have been found in isolates of plasma membrane fractions from lung endothelial cells [41]. Understanding whether and how AKAP12 integrates signalling, mRNA binding and translation roles will provide fundamental and exciting novel insights into membrane-associated protein synthesis.

### Through protein-protein interactions

**Figure 2.**
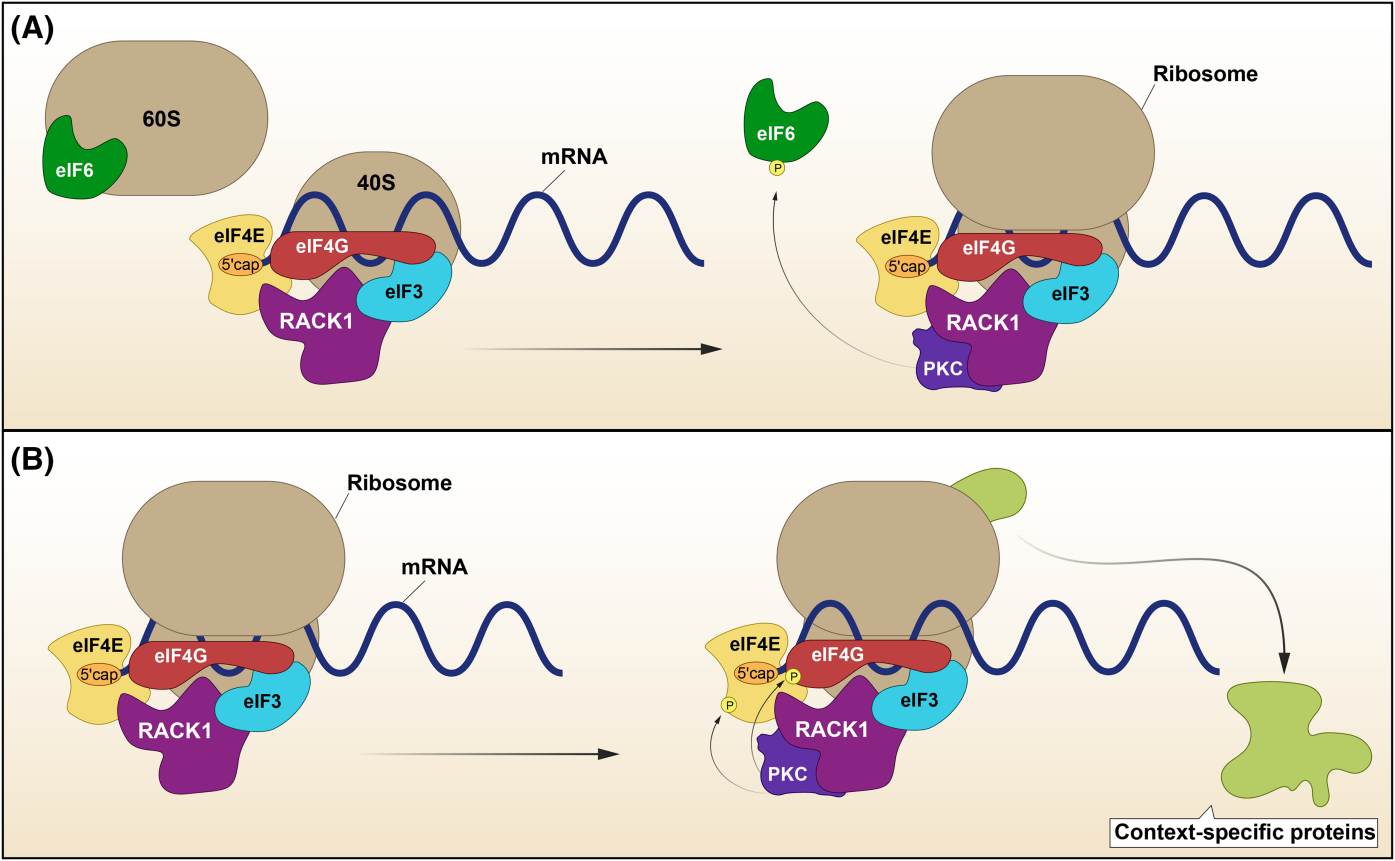
RACK1 participates in translational machinery assembly and function. (**A**) RACK1 recruits PKA to the 40S ribosome subunit to induce the phosphorylation of 60S-associated eIF6. Once phosphorylated, eIF6 is released from 60S to allow ribosome biogenesis. (**B**) RACK1 can also recruit factors such as eIF4E and eIF3 to the initiation complex and induce eIF4E and eIF4G phosphorylation via PKA. P, phosphorylation.

Scaffolding proteins can also input into protein synthesis when acting as platforms for the modulation of translational regulators. The highly conserved receptor for activated C kinase 1 (RACK1) is the most studied scaffold in this context. Since its discovery as an anchoring protein for PKC isozymes, a large number of RACK1 interacting proteins have been documented [42–44]. The scaffolding activity of RACK1 is mediated via its seven WD40 repeats arranged into a β-propeller motif, which is commonly found in proteins that work as anchors for complex assembly. Inhibition of the RACK1/PKC interaction or down-regulation of RACK1 reduces PKC-induced translation [45]. Furthermore, RACK1 null mice are embryonic lethal around gastrulation stages, and whilst heterozygous animals develop to adulthood, they display growth defects and reduced protein synthesis in response to Insulin [46]. RACK1 has been suggested to enhance or repress translation, a phenomenon likely to be context-dependent [47–51]. The first study proposing an implication in mRNA translation identified RACK1 as a component of the ribosome that co-sedimented with the 40S subunit [52]. Subsequent efforts to map its localisation in the ribosome depicted RACK1 near the mRNA exit channel of the head region of 40S, with most of the β-propeller motif exposed for protein interactions [53,54]. In this position, RACK1 is firmly associated to the ribosome, physically affects the motion of 40S, and consequently impacting translation outputs [55,56]. Furthermore, it serves as a local hub for multiple signalling-controlled aspects of ribosomal biology and translational regulation which have been extensively reviewed elsewhere [43,57]. For example, RACK1 regulates ribosomal assembly by recruiting PKCβΙΙ to 40S. Here, PKCβII phosphorylates Eukaryotic Initiation Factor (eIF) 6 to counteract its actions (Figure 2A). eIF6 binds to the 60S subunit to limit its association with 40S and prevent 80S assembly. Upon stimulation of PKC signalling, RACK1 facilitates PKCβΙΙ phosphorylation of eIF6 resulting in its release from 60S and allowing the association of both ribosome subunits [58]. The regulation of translation by RACK1 via the orchestration of initiation factors is not limited to the regulation of eIF6 behaviour. Recruitment of eIF3 and eIF4E to the ribosome is optimal in the presence of ribosome-bound RACK1 [59,60]. Moreover, RACK1/PKCβII phosphorylates both eIF4E and eIF4G downstream of PKC signalling activation, resulting in enhanced protein synthesis (Figure 2B) [48,61,62].

Evidence of translational repression as the result of RACK1-mediated recruitment of specific partners to ribosomes has also been reported. This is the case of TDP-43, an RBP whose activity at the ribosome may result in loss of eIF4E phosphorylation and protein synthesis inhibition [63]. Another example is AGO2, a component of the microRNA silencing machinery, which was shown to associate with 40S in a RACK1-dependent manner [64], but whether signalling regulates this process is not known.

**Figure 3.**
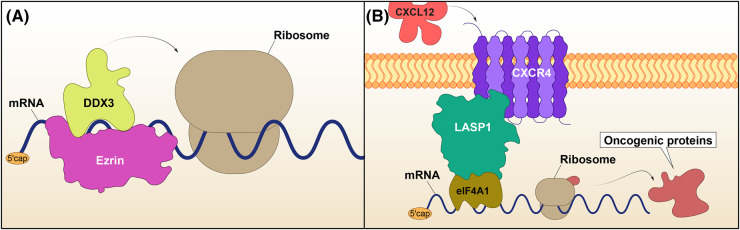
Ezrin and LASP1 interact with RNA helicases. (**A**) The scaffolding protein Ezrin binds DDX3 to regulate its translational functions. (**B**) LASP1 interacts with eIF4A1, a helicase that contributes to translation initiation. This interaction is enhanced by CXCL12-mediated activation of the transmembrane receptor CCXR4, another LASP1 partner. This process is suggested to lead to the translation of mRNAs encoding oncogenic proteins.

Although less understood, scaffolding proteins classically described as cytoskeletal anchors can also bind to regulators of translation and dynamically control their function. Ezrin, Radixin and Moesin (ERM) are related scaffolds that act as linkers between the plasma membrane and F-actin within the cell cortex. The structure of ERM proteins is highly conserved and underpins their cytoskeleton-membrane linkage function. On the N-terminus they contain a FERM domain that interacts with numerous partners, including plasma membrane-associated proteins and other scaffolds . The FERM domain is bridged to the F-actin binding tail of ERM proteins via an α-helix-rich region [65]. Of note, this region has been shown to interact with the regulatory subunits of PKA, placing ERM proteins under the umbrella of AKAPs. In this context, these scaffolds can compartmentalise PKA to subcellular regions where the phosphorylation of signalling substrates at the cell surface regulates cell type-specific functions, from T-cell signalling and axon guidance to placental cytotrophoblast fusion [66–70]. Due to the recruitment of signalling and structural proteins, ERM members have been classically considered key mediators of cytoskeletal rearrangements downstream of cell-surface receptors. Nonetheless, incipient evidence also points to roles in RNA regulation and translation. Whilst we have identified Moesin as part of the RNA-bound proteome in endothelial cells [32], others have revealed that Ezrin is also capable of directly bind to mRNAs [29,30]. In fact, Ezrin co-sediments with monosomes, which at a first instance suggested a potential involvement in translation initiation. Subsequently, efforts to characterise Ezrin partners revealed that this protein interacts with several components of the translational machinery and RBPs [71,72]. In particular, Ezrin was shown to bind to DEAD-box 3 (DDX3), an interaction blocked by a small molecule inhibitor targeting Ezrin and with anti-invasiveness proprieties [72]. DDX3 is an RBP that displays ATPase and helicase activity and modulates translation initiation by resolving secondary 5′UTR mRNA structures, amongst other roles in mRNA biology [73]. Ezrin depletion results in the down-regulation of DDX3 in both hepatocellular and lung cancer cells. In addition, Ezrin can promote the ATPase activity of DDX3, whilst reducing its helicase capacity. For this reason, Ezrin has been proposed to participate in the efficiency of protein synthesis (Figure 3A) [72]. Considering the involvement of Ezrin in recruiting signalling components, how exposure to selective signals could determine Ezrin interactions with translation modulators and control their function remains an open question. Likewise, whether a translation-related function could be linked with its ability to bind RNA is unknown. Due to the ability of Ezrin to compartmentalise PKA, it is tempting to speculate that it may share signalling-translation properties with members of the AKAP family.

The LIM and SH3 protein 1 (LASP1) is another example of a scaffold with an emerging role in translation, as it was found to interact with members of the eIF4F complex [74]. Originally identified as a protein up-regulated during tumourigenesis [75], LASP1 is functionally linked to diverse biological processes, and much of its activity depends on post-translational modifications. Importantly, the phosphorylation of key residues determines dynamic interactions with its many partners [76]. LASP1 interacts with signalling proteins such as CXC chemokine receptors (CXCRs) through its N-terminal LIM domain, which is followed by two F-actin binding Nebulin repeats and a C-terminal SH3 domain [77]. Importantly, phosphorylation of LASP1 at Serine 146 by PKA reduces its affinity to F-actin, but it is necessary for the direct binding to CXCR4 [78,79]. Activation of the receptor by CXC chemokine ligand 12 (CXCL12) reduces phosphorylation at this site and hinders the interaction between LASP1 and CXCR4 [80]. Using an *in vitro* model of triple negative breast cancer, Howard et al. showed that activating the invasiveness-associated receptor CXCR4 with CXCL12 results in the binding of LASP1 to eIF4A1, another helicase that unwinds structures in 5′UTRs and is required for translation initiation (Figure 3B). This was ensued by an increase in the levels of oncogenic proteins, in a LASP1-dependent manner [74]. Whilst the authors clearly placed LASP1 at the centre of a novel signalling-translation axis, how its scaffolding properties contribute to the modulation of the pathway is yet to be fully understood. As signalling induces posttranslational modifications on LASP1 that determine its binding to signalling proteins and the actin cytoskeleton [76,81], understanding how this versatile scaffold may integrate distinct signals, translation and cytoskeletal remodelling would be an interesting avenue to explore.

## Concluding remarks and future outlook

Scaffolding proteins are remarkable hubs for the integration of cellular pathways. Through their interaction with mRNAs and translational regulators, they are incredible platforms where localised protein production can converge with many other molecular events. Novel roles for the scaffolding proteins discussed in this review are waiting to be unveiled. For example, AKAP1-mediated anchoring of an mTOR inhibitor can induce mTOR activation elsewhere in the cell and potentially impact protein production at other sites [82]. Future studies in this direction may reveal a complex network of interconnected translational processes taking place across the cell. Another case is the involvement of AKAP12 in the translation of its target mRNAs at the plasma membrane [32]. Noteworthy, the interaction of transmembrane receptors with ribosomal proteins and RBPs has been reported in neurons, in which ligand-dependent activation of receptors can elicit rapid translation responses [83,84]. Thus, the docking of AKAP12 targets in the vicinity of receptors to facilitate the production of the encoded proteins is an attractive idea, but this remains to be investigated. On another hand, scaffolds that participate in other aspects of mRNA biology may have unappreciated roles in translation. This is the case of AKAP8, mostly associated with mRNA splicing but also implicated in ribosomal RNA biogenesis, a rate-limiting factor in ribosome biogenesis [85]. Cross-talk between scaffolding proteins during localised mRNA translation may also constitute a plausible phenomenon. AKAP12 is known to sequester the Src kinase, supressing its signalling activity at focal adhesions [86], large protein complexes that link the cytoskeleton to the extracellular matrix. Interestingly, RACK1 scaffolding activity is well known to participate in focal adhesion behaviour through interactions with Src [87]. Considering that protein synthesis takes place within the vicinity of focal adhesions [88] and that Src activity can stimulate local mRNA translation [89], it is worth speculating that both scaffolds may act in coordination to control Src at these sites. Finally, RBPs are well understood to undergo changes in their structural and physical properties upon binding to RNAs [90]. Whether such effects on the proteins discussed here could add an extra layer of complexity to the control of translation remains elusive. Although many questions remain unanswered, dissecting the regulation of mRNA translation by scaffolding proteins has uncovered the importance of this fundamental phenomenon. Due to fast-paced technological advances, we anticipate a growing number of studies attributing unappreciated translational properties to more classical scaffolds. Further dissecting the intricacies of their translation-related functions may be supported by synthetic scaffolds, a technology that has been employed to study enzymatic complexes [91]. Undoubtedly, these will offer invaluable new insights into mechanisms of posttranscriptional control of gene expression underpinning biological complexity and diversity.

## Perspectives

- Posttranscriptional control of gene expression is a fundamental process in biology, achieved through the regulation of steps in the lifecycle of mRNAs. Scaffolding proteins can be determinant agents in stages of mRNA translation, with major implications in protein production and consequent cell function.
- A number of proteins with scaffolding activity have been discovered to bind mRNAs and translational regulators, docking them at various subcellular compartments. At their destination, scaffolding proteins work as versatile hubs where diverse molecular events converge to control localised mRNA translation, although this is not understood to the same extent for all examples discussed.
- Continued efforts to explore the remarkable capacity of scaffolding proteins to integrate cellular information will uncover novel models through which they orchestrate posttranscriptional control of gene expression. From new biochemical protocols to innovative bioengineering tools, modern approaches are essential to bring new insights in the regulation of mRNA translation by scaffolding proteins.

## Open Access

Open access for this article was enabled by the participation of Queen's University Belfast in an all-inclusive *Read & Publish* agreement with Portland Press and the Biochemical Society under a transformative agreement with JISC.

## Abbreviations

AKAP: A-kinase anchoring protein
cAMP: cyclic adenosine monophosphate
CXCL: CXC chemokine ligand
CXCR: CXC chemokine receptor
DDX3: DEAD-box 3
eIF: eukaryotic initiation factor
ERM: Ezrin, Radixin and Moesin
F-: filamentous
KH: K-homology
LARP4: La Ribonucleoprotein 4
LASP1: LIM and SH3 protein 1
LPL: lipoprotein lipase
PKA: protein kinase A
PKC: protein kinase C
RACK1: receptor for activated C kinase 1
RBP: RNA binding protein
STAR: Steroidogenic Acute Regulatory Protein
UTR: untranslated region
